# [^18^F]F13640, a 5-HT_1A_ Receptor Radiopharmaceutical Sensitive to Brain Serotonin Fluctuations

**DOI:** 10.3389/fnins.2021.622423

**Published:** 2021-03-08

**Authors:** Matthieu Colom, Benjamin Vidal, Sylvain Fieux, Jérôme Redoute, Nicolas Costes, Franck Lavenne, Inés Mérida, Zacharie Irace, Thibaud Iecker, Caroline Bouillot, Thierry Billard, Adrian Newman-Tancredi, Luc Zimmer

**Affiliations:** ^1^Lyon Neuroscience Research Center, INSERM, CNRS, Université de Lyon, Université Claude Bernard Lyon 1, Lyon, France; ^2^Hospices Civils de Lyon, Lyon, France; ^3^CERMEP-Imagerie du Vivant, Bron, France; ^4^Neurolixis, Castres, France; ^5^Institut National des Sciences et Techniques Nucléaires, Gif-sur-Yvette, France

**Keywords:** [^18^F]F13640, 5-HT_1A_ receptors, PET imaging, serotonin release, agonist, fenfluramine

## Abstract

**Introduction:**

Serotonin is involved in a variety of physiological functions and brain disorders. In this context, efforts have been made to investigate the *in vivo* fluctuations of this neurotransmitter using positron emission tomography (PET) imaging paradigms. Since serotonin is a full agonist, it binds preferentially to G-protein coupled receptors. In contrast, antagonist PET ligands additionally interact with uncoupled receptors. This could explain the lack of sensitivity to serotonin fluctuations of current 5-HT_1A_ radiopharmaceuticals which are mainly antagonists and suggests that agonist radiotracers would be more appropriate to measure changes in neurotransmitter release. The present study evaluated the sensitivity to endogenous serotonin release of a recently developed, selective 5-HT_1A_ receptor PET radiopharmaceutical, the agonist [^18^F]F13640 (a.k.a. befiradol or NLX-112).

**Materials and Methods:**

Four cats each underwent three PET scans with [^18^F]F13640, i.e., a control PET scan of 90 min, a PET scan preceded 30 min before by an intravenous injection 1 mg/kg of d-fenfluramine, a serotonin releaser (blocking challenge), and a PET scan comprising the intravenous injection of 1 mg/kg of d-fenfluramine 30 min after the radiotracer injection (displacement challenge). Data were analyzed with regions of interest and voxel-based approaches. A lp-ntPET model approach was implemented to determine the dynamic of serotonin release during the challenge study.

**Results:**

D-fenfluramine pretreatment elicited a massive inhibition of [^18^F]F13640 labeling in regions known to express 5-HT_1A_ receptors, e.g., raphe nuclei, hippocampus, thalamus, anterior cingulate cortex, caudate putamen, occipital, frontal and parietal cortices, and gray matter of cerebellum. Administration of d-fenfluramine during PET acquisition indicates changes in occupancy from 10% (thalamus) to 31% (gray matter of cerebellum) even though the dissociation rate of [^18^F]F13640 over the 90 min acquisition time was modest. The lp-ntPET simulation succeeded in differentiating the control and challenge conditions.

**Conclusion:**

The present findings demonstrate that labeling of 5-HT_1A_ receptors with [^18^F]F13640 is sensitive to serotonin concentration fluctuations *in vivo*. Although the data underline the need to perform longer PET scan to ensure accurate measure of displacement, they support clinical development of [^18^F]F13640 as a tool to explore experimental paradigms involving physiological or pathological (neurological or neuropsychiatric pathologies) fluctuations of extracellular serotonin.

## Introduction

The 5-HT_1A_ receptor is a subtype of serotonin (5-HT) receptors that belongs to the G-protein coupled receptor family. 5-HT_1A_ receptors couple to G_i/o_ protein and their activation induces an inhibition of cyclase adenylate and a decrease of cAMP synthesis, leading to hyperpolarization of neuron membrane and inhibition of neuron activity (Nichols and Nichols, 2008). 5-HT_1A_ receptors are widely distributed in the central nervous system, being localized in raphe nuclei as somatodendritic receptors (Riad et al., 2000), or in cortical and limbic areas as post-synaptic receptors (Verge et al., 1985; Radja et al., 1991; Miquel et al., 1992). The wide regional expression of 5-HT_1A_ receptors is in accordance with their involvement in many physiological functions, including cognition (Buhot, 1997), cognitive behaviors (Buhot, 1997), pain (Kristian Eide et al., 1990) and in numerous brain disorders such as anxiety (Akimova et al., 2009), depression (Richardson-Jones et al., 2010), schizophrenia (Maćkowiak et al., 2000), Alzheimer’s disease (Truchot et al., 2007), and Parkinson’s disease (Shimizu and Ohno, 2013). In view of the complexity of the functions controlled by 5-HT_1A_ receptors, efforts have been made to investigate them using various positron emission tomography (PET) imaging tools, notably as concerns the fluctuations of endogenous neurotransmitter levels in physiological or pathological conditions. This objective can be pursued by using radiotracers that bind to the target receptors with comparable affinity as the neurotransmitter of interest. Such an approach has been largely applied to the study of the dopaminergic system, using radiotracers of D_2_/D_3_ receptors such as [^11^C]raclopride (Laruelle, 2000). Unfortunately, measuring endogenous 5-HT release using PET has proved more challenging (Paterson et al., 2010; Tyacke and Nutt, 2015). Currently, very few radiotracers display sensitivity to changes in 5-HT levels, including [^11^C]Cimbi-36, a 5-HT_2A_ receptor agonist radioligand (da Cunha-Bang et al., 2019) and [^11^C]AZ10419369, a 5-HT_1B_ receptor agonist radioligand (Yang et al., 2018). As concerns 5-HT_1A_ receptors, some studies were carried out in rodent models with the antagonist [^18^F]MPPF (Zimmer et al., 2002, 2003) but did not show sufficient sensitivity to robustly evaluate physiological changes of endogenous serotonin levels,

The main reason cited for the lack of sensitivity of 5-HT_1A_ radiopharmaceuticals is the fact that they are antagonists or, at best, partial agonists (Colom et al., 2019). *In vitro* studies indicate that antagonist ligands bind similarly to receptors in both coupled and uncoupled states (Laruelle, 2000). Since serotonin is a full agonist which binds preferentially to G-protein-coupled receptors, competition with a PET antagonist is therefore “diluted” by the latter’s additional interaction with uncoupled receptors. In this context, using agonist radiotracers seems a more promising strategy, as they directly compete with endogenous serotonin on the same G-protein-coupled sites. Accordingly, [^11^C]Cimbi-36 shows a higher sensitivity to serotonin fluctuations compared to the antagonist radiotracer, [^18^F]altanserin (Jørgensen et al., 2017).

We previously evaluated a series of full-agonist ligands of 5-HT_1A_ receptors, F13714, F15599 (a.k.a. NLX-101) and F13640 (a.k.a. befiradol or NLX-112), as potential radiotracers. Although the labeling of F13714 and F15599 with fluorine-18 was successful, their first use *in vivo* as radiotracers showed that the signal to noise ratio was too low for [^18^F]F15599, and that [^18^F]F13714 binding appeared to be irreversible (Lemoine et al., 2010, 2012). In contrast, [^18^F]F13640 showed satisfying properties for neuroimaging. PET studies in rats, cats and non-human primates revealed that it specifically targets 5-HT_1A_ receptors, and showed a good signal to noise ratio (Vidal et al., 2018a). Moreover, *ex vivo* autoradiography studies in rat showed that [^18^F]F13640 was almost ten times more sensitive to competition binding with serotonin than the antagonist [^18^F]MPPF (Vidal et al., 2018a), thus justifying the current study.

The aim of the present PET study was therefore to further investigate the PET sensitivity of [^18^F]F13640 to changes in endogenous serotonin levels induced by 1 mg/kg of d-fenfluramine, a serotonin releaser. Studies were conducted in cat due to its higher imaging resolution than mice and rats (Aznavour et al., 2006), as shown in our previous experiments (Vidal et al., 2018a,b). We performed two type of pharmacological PET protocols with [^18^F]F13640: (i) a d-fenfluramine pretreatment 30 min before PET acquisition (5-HT blocking paradigm); and (ii) a challenge of d-fenfluramine 30 min after start of PET acquisition (5-HT displacement paradigm). Additional *in vitro* autoradiography experiments were also carried out and the data were analyzed using a ROI-based and a voxel-based approaches, with simple estimations of 5-HT_1A_ receptors occupancy by serotonin or kinetic modeling of serotonin release using the lp-ntPET protocol (Normandin et al., 2012).

## Materials and Methods

### [^18^F]F13640 Radiosynthesis

Synthesis of [^18^F]F13640 and quality controls pathways were previously described (Vidal et al., 2018a). Briefly, after production of fluoride preparation ^18^O(p,n)^18^F cyclotron reaction, 5 mg of F13640 nitro precursor are introduced. After nucleophile substitution, [^18^F]F13640 is obtained by separation on a preparative HPLC column (SymmetryPrepC18, 7 μm, 7.80 mm × 300 mm, Waters). The radiotracer is formulated *via* solid phase extraction techniques using a Sep-Pak Light C18 cartridge (Waters). The final product is eluted with 1 mL of ethanol, diluted with saline and finally sterilized by filtration (sterile filter Millex-GS, 0.22 μm; Millipore).

### Autoradiography Studies

The brain of one cat was extracted after euthanasia obtained by short inhalation of isoflurane and in accordance with European guidelines for care of laboratory animals (2010:63:EU). The brain was immediately frozen in 2-methylbutane cooled with dry ice (−29°C). Coronal sections (30 μm thick) were cut using a −20°C cryostat, thaw-mounted on glass slides, and allowed to air dry before storage at −80°C until used. At the day of the experiment, all slides were incubated for 20 min in Tris phosphate-buffered saline buffer, pH 7.5, containing 37 kBq/ml of [^18^F]F13640 (F13640 1 μmol/L). For competition studies, slices were placed in the same buffer plus four different concentrations of serotonin (1 nM, 2 nM, 5 nM, and 10 nM). For coupling studies, 10 μM of Gpp(NH)p, a non-hydrolysable analog of guanosine 5′-triphosphate, was added. After incubation, slides were dipped in distilled cold water (4°C) and then dried and juxtaposed to a phosphor imaging plate and scanned for 60 min (BAS-1800 II; Fujifilm). Regions of interest (ROIs) were manually drawn using Multigauge software (Fujifilm). The results were expressed in optical densities (PSL/mm^2^).

### PET Studies

#### Animals and Procedures

Four male cats (Isoquimen S.L., Barcelona, Spain) weighting 3.5–5.5 kg underwent PET scans in 12 separate sessions. All experiments were performed in accordance with European guidelines for care of laboratory animals (2010:63:EU). Before each exam, cats underwent a premedication with medetomidine (30–60 μg/kg subcutaneous) followed by anesthesia induction using intramuscular injection of 30 μg/kg medetomidine plus 2 mg/kg ketamine. Radiotracer injection was ensured by a catheter insertion into the cephalic vein of the forearm continuously perfused by NaCl 9%. Endotracheal intubation was performed to ensure a respiration rate of 15 breaths/min and anesthesia was maintained by constant insufflation of 2% isoflurane. Heart rate and SpO2 were continuously monitored. Cats were placed in ventral decubitus in an acrylic stereotactic apparatus with ear bars. Body temperature was maintained using a heated water blanket.

#### Study Design

Each cat underwent three PET acquisitions, i.e., one control acquisition, one d-fenfluramine intravenous pretreatment acquisition, and one challenge acquisition consisting in d-fenfluramine injected intravenously during acquisition. The agonist radiotracer of 5-HT_1A_ receptors [^18^F]F13640 was injected in a bolus at the start of the PET acquisition over 30 s (108 ± 19 MBq), diluted in 1 mL of NaCl 0.9%. D-fenfluramine was administered at 1 mg/kg diluted in 1 mL of NaCl 0.9%, 30 min before PET acquisition, for the pre-treatment study, and 30 min after radiotracer injection, for the pharmacological challenge. The corresponding control experiments consisted in a 1 mL saline administration 30 min after the radiotracer injection.

#### Data Acquisition and Reconstruction

Images were acquired on a PET/CT Biograph mCT (Siemens) at the CERMEP-imaging platform. Before PET emission scan, a rapid CT acquisition was performed to compute a brain attenuation map. The PET emission scan was performed for 90 min in list mode immediately after intravenously injection of [^18^F]F13640. A dynamic PET image was reconstructed in a series of 28 sequential frames (4 × 30 s; 4 × 60 s; 8 × 180 s and 12 × 300 s). PET images were reconstructed with a fully three-dimensional (3D) ordinary Poisson OSEM reconstruction (OP-OSEM). PET data were preprocessed using MINC Toolkit (McConnell Brain Imaging Centre, Montreal, QC, Canada) and modeled with programs of the Turku PET Center library. For each cat, a PET sum image was computed and used as target for the warping of a multi-subject MRI template (Lancelot et al., 2010), which allows the parcellation of the brain into 20 anatomical brain regions of interest. In addition, a bisymmetrical anterior part of centrum semiovale was manually drawn on display software and was considered as a reference region. Time-activity curves in kBq/cc were extracted for each ROI, bilaterally averaged. Each time point of the TAC was converted into binding ratios compared to the centrum semiovale, which was chosen as region of reference.

### PET Data Analysis

#### Occupancy Analysis in Pretreatment and Challenge Studies

In pretreatment experiments, averaged binding ratios between 40 and 88 min were compared with the control experiments by Student *t*-tests (*p* < 0.01; non-corrected; GraphPad Prism 6). In the pretreatment study, blocking rates in the frame [40;88] min were calculated as below:

$$Blockingrate\left(\%\right)=\frac{R\,a\,t\,i\,o\,\left(C\,o\,n\,t\,r\,o\,l\right)-R\,a\,t\,i\,o\,\left(P\,r\,e\,t\,r\,e\,a\,t\,m\,e\,n\,t\right)}{R\,a\,t\,i\,o\,\left(C\,o\,n\,t\,r\,o\,l\right)-1}\times 100$$

In the pharmacological challenge experiments, post-injection binding ratios between 40 and 88 min were compared with pre-injection binding ratios between 15 and 30 min to estimate occupancy rates (Zhang and Fox, 2012):

$$Occupancy\left(\%\right)=\frac{R\,a\,t\,i\,o\,\left(P\,r\,e-i\,n\,j\,e\,c\,t\,i\,o\,n\right)-R\,a\,t\,i\,o\,\left(P\,o\,s\,t-i\,n\,j\,e\,c\,t\,i\,o\,n\right)}{R\,a\,t\,i\,o\,\left(P\,r\,e-i\,n\,j\,e\,c\,t\,i\,o\,n\right)-1}\times 100$$

The occupancy values obtained for each scan were averaged and compared to the control condition by Student *t*-tests (*p* < 0.01; non-corrected; GraphPad Prism 6). The occupancy rates in challenge condition were subtracted with the occupancy rates calculated in the control condition to finally obtain a corrected occupancy rates, taking into account non-specific changes that could occur without any challenge.

#### Voxel-Based Analysis of the Pharmacological Challenge Study

Ratio images were generated by dividing the PET signal in each voxel by the mean signal in the centrum semiovale using the Turku PET Center software (*Imgratio* function) to generate one baseline image from 15 to 30 min and one global post injection image from 30 to 90 min. These ratio images were smoothed using a 1 mm × 1 mm × 1 mm isotropic Gaussian filter, spatially normalized in the template space, and statistically analyzed with SPM 12. Pooling the four subjects, a statistical map of the significant decreases of [^18^F]F13640 binding after the pharmacological challenge was computed with two successive paired *t*-test, the first performed for each scan using the relative contrast (Baseline image – Post injection), the second to compare the saline and challenge conditions, corresponding to the final contrast [(Baseline image – Post injection)_challenge_ – (Baseline image – Post injection)_saline_] for each post-injection time interval. Statistical significance was set at *p* < 0.01 uncorrected.

#### Kinetic Modeling of [^18^F]F13640 Displacement in the Pharmacological Challenge Study

We applied the lp-ntPET model (Normandin et al., 2012) optimized with the 2-step method (Merida et al., 2018) on control and challenge conditions in order to characterize the transient tracer displacement induced by an endogenous serotonin release. This model computes the perfusion ratio of the ROI relative to the reference region (R1), the efflux rate in the reference region (k_2_), and in the ROI (k_2a_), as well as four parameters describing the analytical curve of the endogenous serotonin discharge (modeled as a gamma variate function) underlying the TAC curve decrease after pharmacological challenge. The discharge equation parameters are, start time of the discharge (t_D_), time of the peak of the discharge (t_P_), a parameter controlling the shape of the discharge (α), and the amplitude of the discharge (γ). The following constraints were applied on parameters: t_D_ was searched between 30 and 50 min with a step of 1 min, t_P_ was searched between 31 and 90 min with a step of 1 min, and α ranged from 0.5 to 10 with a step of 0.5.

Curves of k_2a_ variation across time (expressed as the % of baseline) were calculated to highlight dynamic changes in regional 5-HT receptors (Kyme et al., 2019). Receptor occupancies were obtained, in each region, from the dynamic BP_ND_ (DBP_*ND*_) (Sander et al., 2013) estimated with the 2-step lp-ntPET model.

$$Occupancylp-ntPET\left(\%\right)=\frac{D\,B\,P_{N\,D}\,\left(B\,a\,s\,e\,l\,i\,n\,e\right)-D\,B\,P_{N\,D}\,\left(P\,o\,s\,t\,i\,n\,j\,e\,c\,t\,i\,o\,n\right)}{D\,B\,P_{N\,D}\,\left(B\,a\,s\,e\,l\,i\,n\,e\right)}\times 100$$

Where DBP_*ND*_ (baseline) is the averaged DBP_*ND*_ on the interval 20–30 min and DBP_*ND*_ (post injection) is the averaged DBP_*ND*_ on the interval 40–90 min.

## Results

### Radiosynthesis

Radiolabeling of the nitro-precursor with fluorine achieved a radiochemical yield of 6% corrected for decay and 90 min-radiosynthesis time. The synthesis led to no other radioactive derivatives and the chosen HPLC conditions ensured good separation of [^18^F]F13640 from its precursor (Vidal et al., 2018a). Radiochemical purity was higher than 98%, and specific activity at EOS ranged between 25 and 124 GBq/μmol. Radioactivity injected ranged from 76 to 137 MBq.

### *In vitro* Studies

**FIGURE 1:**
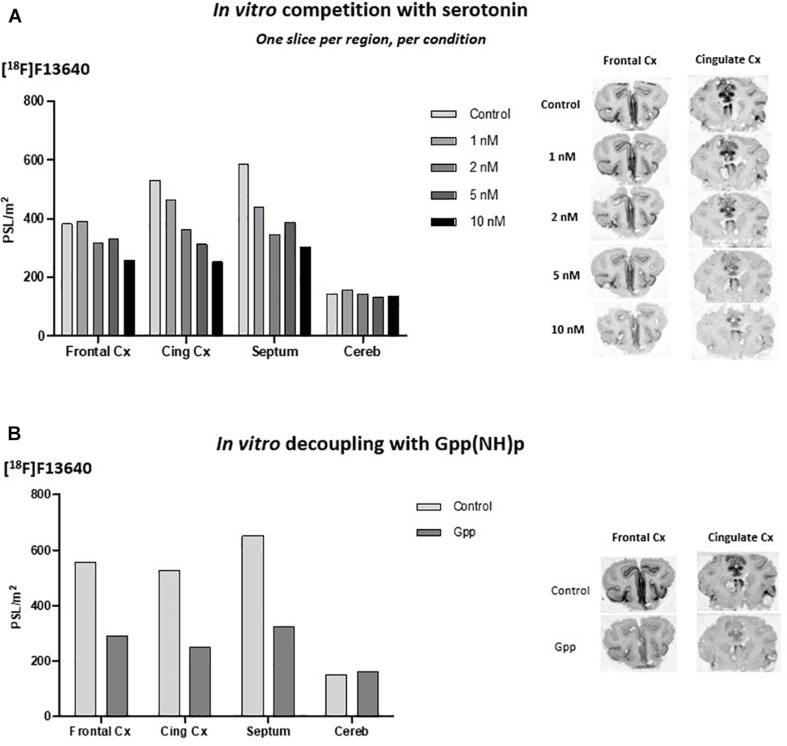
**(A)** [^18^F]F13640 *in vitro* binding in cat brain with increasing concentrations of serotonin (Front Cx, frontal cortex; Cing, cingulate cortex; Septum, lateral septum; Cereb, cerebellum). **(B)** [^18^F]F13640 *in vitro* binding in cat brain with addition of 10 μM of Gpp(NH)p (Front Cx, frontal cortex; Cing Cx, cingulate cortex; Septum, lateral septum; Cereb, cerebellum).

*In vitro* sensitivity of [^18^F]F13640 was evaluated by incubation of cat brain slices with increasing concentrations of serotonin in buffer. Binding was decreased in a concentration-dependent manner especially for the cingulate cortex (52% inhibition at 10 nM), the frontal cortex (32% at 10 nM), and the lateral septum (48% at 10 nM) (Figure 1A). No changes were observed in the cerebellum. Incubation of cat brain slices with 10 μM of Gpp(NH)p, a receptor/G-protein uncoupling agent, induced significant reduction of [^18^F]F13640 binding in comparison with control experiment: 52% in the cingulate cortex, 48% in the frontal cortex, 50% in lateral septum and no changes in the cerebellum (Figure 1B).

### *In vivo* Distribution of [^18^F]F13640

**FIGURE 2:**
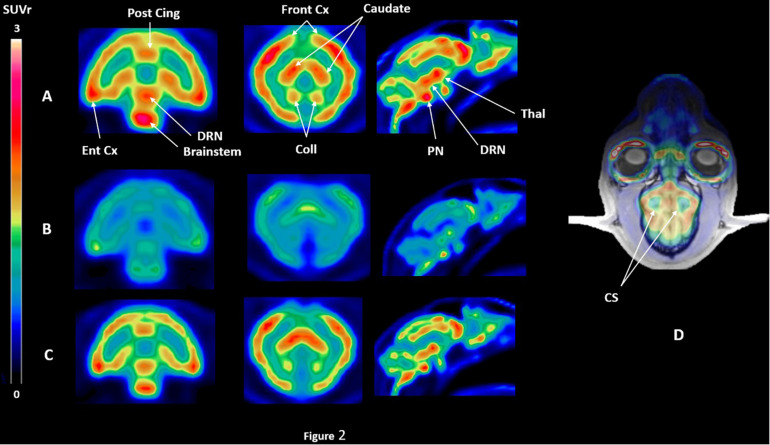
SUVr PET images of [^18^F]F13640 binding in the three experimental conditions for one cat, i.e., **(A)** control conditions, **(B)** pre-injection of d-fenfluramine 30 min before PET acquisition, using the centrum semiovale as a reference region **(D)**. SUVr images are averaged from 32 to 90 min time of acquisition; **(C)** injection of d-fenfluramine 30 min after PET acquisition beginning (Post cing, posterior cingulate cortex; Front Cx, frontal cortex; Caudate, caudate putamen; Ent Cx, entorhinal cortex; DRN, dorsal raphe nucleus; PN, pontine nucleus; Thal, thalamus; Coll, colliculi; CS, centrum semiovale).

**FIGURE 3:**
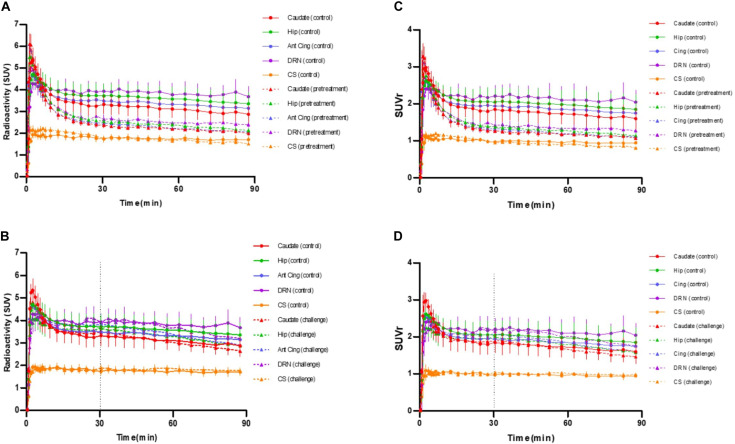
**(A)** Averaged time-activity curves of [^18^F]F13640 ± SEM (circles, control conditions; triangles, pretreatment study with a pre-injection of 1 mg/kg of d-fenfluramine, 30 min before PET acquisition). **(B)** Averaged time-activity curves of [^18^F]F13640 ± SEM (circles, control conditions; triangles, challenge study with a post-injection of 1 mg/kg of d-fenfluramine, 30 min after beginning of PET acquisition). **(C)** Averaged SUVr of [^18^F]F13640 ± SEM (circles, control conditions; triangles, pretreatment study with a pre-injection of 1 mg/kg of d-fenfluramine, 30 min before PET acquisition). **(D)** Averaged SUVr of [^18^F]F13640 ± SEM (circles, control conditions; triangles, challenge study with a post-injection of 1 mg/kg of d-fenfluramine, 30 min after beginning of PET acquisition) Caudate, caudate putamen; Hip, hippocampus; Cereb, cerebellum; Ant Cing, anterior cingulate cortex; DRN, dorsal raphe nucleus; CS, centrum semiovale.

[^18^F]F13640 kinetics showed rapid uptake in the whole cat brain, and a slow wash-out during the 90 min of acquisition (Figures 2, 3). The dorsal raphe nucleus (DRN), the anterior cingulate cortex, the hippocampus, and the thalamus showed the higher uptake values. The cortical parts of the cerebellum showed an intermediate uptake unlike the cerebellar nuclei. We also identified the anterior part of centrum semiovale as a region displaying a very low uptake (Figure 2). Given that this region was unlikely to be impacted by partial volume effects compared to the cerebellar nuclei (due to their proximity with the cortical parts displaying high uptake) and that the signal was low and constant in the three conditions (Figure 3), this region was chosen as a reliable reference region.

### Pretreatment Study With D-Fenfluramine

The intravenous administration of d-fenfluramine (1 mg/kg) 30 min before radiotracer injection induced a drastic decrease of [^18^F]F13640 uptake (Figure 2). Significant decreases of binding ratios control occurred in caudate, thalamus, hippocampus, anterior cingulate cortex, frontal cortex, parietal cortex, and DRN. The average blocking rate varied between 96% (gray matter of the cerebellum) and 33% (pontine nuclei) (Table 1).

**TABLE 1:** 5-HT_1A_ receptor occupancy rate calculated for pretreatment study, challenge study (standard analysis and lp-ntPET modeling) and *p*-value of Wilcoxon paired test comparing occupancy computed with BP or with lp-ntPET.

	**Caudate**	**Thal**	**Hip**	**Cereb**	**Ant Cing**	**Front Cx**	**Occ Cx**
**Pretreatment study**							
Cat 1	67.7	59.2	64.9	94.7	68.7	73.7	93.4
Cat 2	47.4	33.9	45.7	77.2	50.1	44.3	71.4
Cat 3	41.1	30.3	40.3	93.2	47.1	50.2	57.3
Cat 4	67.6	66.5	76.2	95.5	73.8	83.7	86.1
Blocking rate (% ± sd)	56.0 ± 13.8	47.5 ± 18.1	56.8 ± 16.7	90.2 ± 8.7	59.9 ± 13.3	63.0 ± 18.8	77.1 ± 16.0
**Challenge study**							
Cat 1	17.5	10.8	18.5	31.7	20.5	15.9	26.4
Cat 2	16.2	12.1	15.1	30.0	19.8	16.2	23.5
Cat 3	19.1	5.5	11.8	32.7	12.9	6.6	23.8
Cat 4	15.6	13.1	18.0	31.1	21.0	16.6	24.8
Occupancy* (% ± sd)	17.1 ± 1.5	10.4 ± 3.4	15.8 ± 3.1	31.4 ± 1.2	18.5 ± 3.8	13.8 ± 4.8	24.6 ± 1.3
**Lp-ntPET**							
Cat 1	11.2	11.0	15.6	15.7	9.1	11.0	16.0
Cat 2	11.9	14.0	11.7	16.3	15.8	13.6	14.8
Cat 3	12.2	12.0	16.2	16.9	11.0	9.0	14.9
Cat 4	10.8	14.0	12.5	15.5	15.3	14.8	10.9
Occupancy lp-ntPET*(% ± sd)	11.5 ± 0.6	12.8 ± 1.5	14.0 ± 2.2	16.1 ± 0.6	12.8 ± 3.3	12.1 ± 2.6	14.2 ± 2.2
*p*-value	0.125	0.125	0.625	0.125	0.125	0.375	0.125

*Occupancy rate were adjusted on control condition for challenge study (Caudate, caudate putamen; Thal, thalamus; Hip, hippocampus; Cereb, cerebellum; Ant Cing, anterior cingulate cortex; Front Cx, frontal cortex; Temp Cx, temporal cortex; Occ Cx, occipital cortex).

### Challenge Study With D-Fenfluramine

**FIGURE 4:**
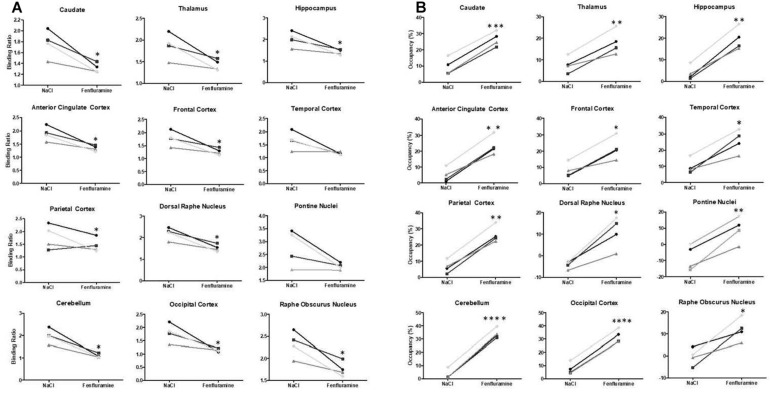
**(A)** Averaged binding ratios of [^18^F]F13640 calculated in control (NaCl) and pretreatment (d-fenfluramine) conditions in the four cats (^∗^*p* < 0.05; ^∗∗^*p* < 0.01; ^*⁣*⁣**^p < 0.0001 paired *t*-test, non-corrected). **(B)** Occupancy (%) of serotonin calculated in control (NaCl) and challenge (d-fenfluramine) conditions in the four cats (^∗^*p* < 0.05; ^∗∗^*p* < 0.01; ^*⁣*⁣**^p < 0.0001). For values see Table 1.

**FIGURE 5:**
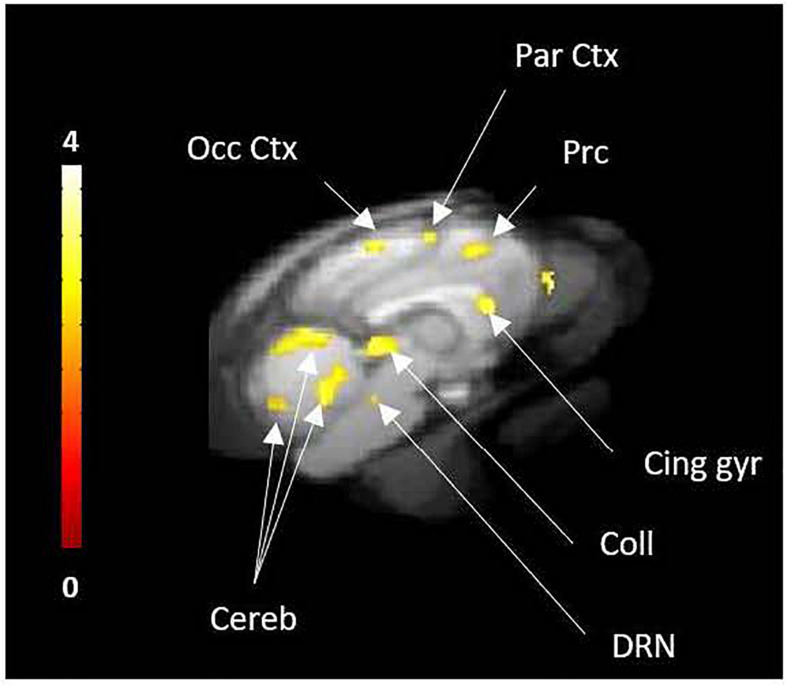
Voxel-to-voxel analysis from 30 to 90 min PET acquisition, showing the significant decreases of [^18^F]F13640 binding after d-fenfluramine challenge compared to control scans (*p* < 0.01, non-corrected). Occ Ctx, Occipital Cortex; Par Ctx, Parietal Cortex; Cing gyr, Cingulate gyrus; Coll, Colliculi; DRN, Dorsal raphe Nuclei; Cereb, Cerebellum; Prc, precuneus.

The intravenous injection of d-fenfluramine (1 mg/kg), 30 min after radiotracer injection, slightly decreased the time activity curves/increased the wash-out rate of [^18^F]F13640. The impact of d-fenfluramine on TAC slope started at 40 min (Figure 3). Significant changes in occupancy rate were observed in cerebellum, hippocampus, caudate, occipital cortex, parietal cortex, and thalamus. The average corrected rate of occupancy ranged from 10% for the thalamus to 31% for the cerebellum (Figure 4). The voxel analysis underlined a progressive effect of the competition between the radiotracer and 5-HT induced by d-fenfluramine. Regions statistically concerned by this effect (*p* < 0.01) were rostral colliculus, cerebellar gray matter, raphe nuclei, marginal gyrus, precuneus, cingulate gyrus, and lateral septum (Figure 5).

### Kinetic Modeling With 2-Step lp-ntPET

**FIGURE 6:**
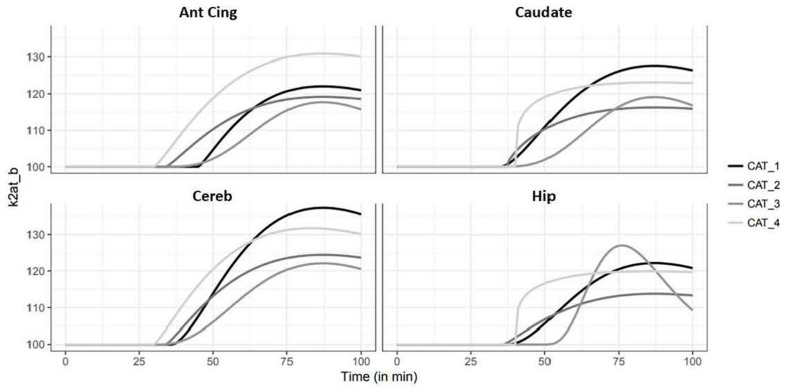
Simulation of the kinetics of serotonin release illustrated by variation (%) of the efflux rate k2at_b of four regions of interest (Ant Cing, anterior cingulate cortex; Caudate, caudate putamen; Cereb, cerebellum; Hip, hippocampus).

Occupancy rates estimated using the 2-step lp-ntPET model displayed high displacement of the tracer in cerebellum, hippocampus, and the cingulate, frontal, temporal and occipital cortices, ranging from 12% in caudate to 16% in cerebellum (Figure 4). Interestingly, the curves of estimated parameter k2a displayed slight differences among animals and regions of interest. For cat 1, the shape of the curve was similar for all regions, with the detected start time of discharge at 15 min and a peak at 45–50 min after d-fenfluramine administration. Cat 2 displayed similar curves with a lower peak amplitude. For cat 3 the curves were slightly different, with a start time occurring later, at around 20 min, and a peak at 50 min after d-fenfluramine administration in the anterior cingulate cortex, cerebellum and caudate. In the hippocampus, the curve was even sharper with a start time at 25 min and a peak at 45 min after the challenge. For cat 4, two different kinetics were observed. In the cerebellum and the anterior cingulate cortex, the start time occurred very early, just after d-fenfluramine administration, followed by a slow increase to a peak reached between 45 and 50 min. In the caudate and the hippocampus, the start time was about 10 min after the challenge and k2a values increased rapidly, reaching a plateau 30 min after the challenge. We also noticed variations in terms of peak amplitude among regions (anterior cingulate cortex: cat4 > cat1 > cat2 > cat3; caudate: cat1 > cat4 > cat3 > cat2; cerebellum: cat1 > cat4 > cat2 > cat3; hippocampus: cat3 > cat1 > cat4 > cat2) and among cats (cat1: cerebellum > caudate > anterior cingulate cortex > hippocampus; cat2 and cat4: cerebellum > anterior cingulate cortex > caudate > hippocampus; cat3: hippocampus > cerebellum > caudate > anterior cingulate cortex). The amplitude of peak ranged from 110 to 140% of k2a basal values (Figure 6). Finally, a Wilcoxon Rank paired test showed that there is no statistical difference comparing occupancy computed with BP or with lp-ntPET for any of the regions (Table 1).

## Discussion

In certain controlled paradigms, PET imaging enables the *in vivo* measure of neurotransmitter fluctuations in the living brain. For example, the determination of changes in extracellular dopamine levels elicited by dopamine-releasers has been well described, but equivalent exploration of serotonin neurotransmission has proven to be difficult (Laruelle, 2000; Zimmer et al., 2002, 2003; Paterson et al., 2010; Tyacke and Nutt, 2015; Yang et al., 2018; Colom et al., 2019; da Cunha-Bang et al., 2019). Several teams have attempted to identify and characterize serotoninergic radiotracers that are sufficiently sensitive to detect changes in endogenous serotonin concentrations following an acute pharmacological challenge, but with limited success. Recently, a European consortium demonstrated that [^11^C]CIMBI-36 binding is sensitive to increased levels of brain serotonin release following a d-amphetamine challenge (Erritzoe et al., 2020). In the present preclinical study, we propose an alternative radiopharmaceutical with a number of advantages. On the one hand, [^18^F]F13640 binds widely to 5-HT_1A_ receptors in the brain and covers many brain regions in both animals (Vidal et al., 2018a) and, as recently shown, in humans (Colom et al., 2020). On the other hand, this radiopharmaceutical is radiolabeled with fluorine 18, allowing prolonged experimental protocols as well as broader dissemination to other research sites.

As previously mentioned, the pharmacological characteristics of F13640 (a highly specific 5-HT_1A_ receptor agonist, a.k.a. NLX-112 or befiradol) and promising *in vitro* results prompted us to initiate this preclinical proof-of-concept study.

### Effect of D-Fenfluramine on Brain Serotonin Levels

The choice of d-fenfluramine, a well-described 5-HT releaser, was justified by its very low affinity for 5-HT_1A_ receptors (K_i_ = 831 nM), ruling out the possibility of a direct competition with [^18^F]F13640 at administered doses (33). Although the binding profile of d-fenfluramine is well-known, there is a lack of data concerning effects of d-fenfluramine in different brain regions in terms of 5-HT release in cat. One study reported that basal serotonin concentrations in dorsal raphe of cats ranged from 0.32 nM during REM sleep to 0.8 nM during waking (Portas and McCarley, 1994). In the anesthetized rat, previous data indicated basal levels of serotonin of 0.24–0.25 nM in the ventral hippocampus (Assié and Koek, 1996). The intraperitoneal injection of 10 mg/kg of d-fenfluramine increased 5-HT levels to a maximum of about 7 nM (that is, nearly 3000% of baseline levels) after 40 min. In rat striatum, basal levels of serotonin were reported at 0.11 nM, with 1 mg/kg of d-fenfluramine ip increasing the concentration to 282% of basal levels after 2 h in conscious animals (Balcioglu and Wurtman, 1998). Basal levels in frontal cortex were estimated at 1.2 nM and administration of 10 mg/kg d-fenfluramine induced an increase to 420% of the baseline 30 min after ip injection (Gardier et al., 1994). Hume et al. (2001) reported that, after 40 min, d-fenfluramine 10 mg/kg ip increased 5-HT levels 4.5 fold in frontal cortex and 15 fold in hippocampus. In pig, a d-fenfluramine dose of 0.5 mg/kg induced an increase of 1123% of serotonin in medial prefrontal cortex, 15 min after intravenous injection (Jørgensen et al., 2017). These results and others show heterogenous basal levels of serotonin among brain regions and a widespread effect of d-fenfluramine, with some variability depending on the regions and species considered. A major limitation of many of these microdialysis studies is that high doses of d-fenfluramine can lead to large increases in extracellular serotonin concentrations that may not reflect normal-physiological conditions. In view of these multiple experimental results, the present study therefore used a modest dose of fenfluramine in order to mimic physiological levels of serotonin fluctuation.

### Effect of D-Fenfluramine on PET Radiotracers Binding

Measuring endogenous levels of serotonin by competition with a PET radiotracer is a real challenge and no 5-HT_1A_ receptor radiotracer has previously been successfully used for this purpose. The development of such a radiotracer faces many challenges related to both its own kinetic properties and to the characteristics of the serotonin system (Paterson et al., 2010). For instance, the *in vivo* density of 5-HT_1A_ receptors in the high affinity (i.e., G-protein-coupled) state is considered to be low, as suggested by *in vitro* findings, which may explain the lack of sensitivity of antagonist radiotracers to changes in 5-HT levels: serotonin would compete with a very small portion of the total binding pool of such tracers (Nénonéné et al., 1994; Udo de Haes et al., 2005). This was also suggested by *in vivo* PET studies where agonists only show occupancies of 10–20% of the [^18^F]antagonist-labeled sites (Bantick et al., 2004). Numerous attempts with other 5-HT_1A_ radiotracers to demonstrate changes of binding induced by pretreatment or challenge with d-fenfluramine showed contrasted results. While pretreatment with d-fenfluramine at 10 mg/kg ip (Hume et al., 2001) and challenge studies with d-fenfluramine at 10 mg/kg iv in rats induced a significant decrease of [^11^C]WAY100635 uptake in hippocampus (19), iv infusion of 5 mg/kg and 10 mg/kg of d-fenfluramine in conscious non-human primates did not induce significant changes in [^18^F]MPPF binding (Udo de Haes et al., 2006). By contrast, [^18F^]MPPF was sensitive to d-fenfluramine challenge in a dose dependent manner in anesthetized rats (Zimmer et al., 2002). For [^11^C]CUMI-101, a pre-injection with 2.5 mg/kg of d-fenfluramine and 2–4 mg/kg of citalopram iv in baboon was responsible for a binding decrease of 15–30% across brain regions (Milak et al., 2010). In humans, one study showed no changes in binding potential of this radiotracer after infusion of 0.15 mg/kg of citalopram (Pinborg et al., 2012) whereas another study showed few increases of binding potential at post-synaptic areas after infusion of 10 mg of citalopram (Selvaraj et al., 2012). No significant evidence of sensitivity to 5-HT release was shown for [^18^F]FCWAY and [^18^F]FPWAY (Jagoda et al., 2006).

### Pretreatment Study With D-Fenfluramine

All these previous studies contrasted with our preliminary findings with the full agonist [^18^F]F13640. *Ex vivo* autoradiography studies in rat brain revealed a significant binding reduction following d-fenfluramine iv pre-administration at doses of 0.5 mg/kg, 1 mg/kg, and 5 mg/kg in hippocampus, anterior cingulate cortex and dorsal raphe whereas only a tendency was observed for [^18^F]MPPF at in the same conditions (Vidal et al., 2018a). These results suggested that [^18^F]F13640 is very sensitive to changes in 5-HT levels, especially in view of the fact that the doses of d-fenfluramine needed for a significant effect were ten times lower than those used for [^18^F]MPPF. For the present study, we therefore chose to use a dose of 1 mg/kg of d-fenfluramine, which is lower than the doses used in previous studies investigating the sensitivity of 5-HT_1A_ radiotracers but that were anticipated to be high enough to induce strong decreases of [^18^F]F13640 binding. As expected, 1 mg/kg of d-fenfluramine administered in pretreatment or a challenge induced marked changes in [^18^F]F13640 labeling kinetics (Figures 2, 3). The pre-administration of d-fenfluramine induced a highly significant decrease of agonist radiotracer signal, and the blocking rate calculated was higher for hippocampus, anterior cingulate cortex and dorsal raphe in comparison with the previous *ex vivo* autoradiography study (58%, 60%, and 55%, respectively) (Vidal et al., 2018a). The gray matter of the cerebellum was the most impacted region (90%), see discussion below.

### Challenge Study With D-Fenfluramine

In this series of experiments, where d-fenfluramine was injected 30 minutes after the radiotracer, we did not observe pronounced effects as for the previous pretreatment studies (Figure 3). This was expected, given the slow dissociation rate of this radiotracer (Heusler et al., 2010), due to its high affinity for 5-HT_1A_ receptors, combined with the short PET acquisition time of 90 min. Another mechanism that could contribute to the differences in absolute occupancy values between pretreatment and challenge studies would be a massive internalization of receptors after pretreatment studies, leading to a low density of high affinity sites at the surface of neurons (Zimmer et al., 2004; Ginovart, 2005). However, this hypothesis seems unlikely because time activity curves displayed a slope inflection 10 min after serotonin release induced by d-fenfluramine in almost all regions of interest except the centrum semiovale. Although occupancy by 5-HT was lower when d-fenfluramine was administered 30 min after ^18^F-F13640, it was significantly different from control scans in all regions of interest, with values ranging from 10% in the thalamus to 31% in the gray matter of cerebellum. Interestingly, the magnitude of the occupancy is in the same range of those calculated for 5-HT_1B_ receptors with the partial agonist [^11^C]AZ10419369, i.e., between 4 and 27% at 1 mg/kg of fenfluramine in three monkeys (Finnema et al., 2012) and also for the [^11^C]CUMI-101 on 5HT_1A_ receptors, i.e., 24% with 2.5 mg/kg fenfluramine in baboons (Milak et al., 2010). Accordingly, the voxel-based analysis detected significant decreases of [^18^F]F13640 in many regions, including the dorsal raphe and in several serotonergic projection areas (precuneus, cingulate, parietal and occipital cortices, colliculi, and cerebellar cortex).

### Changes of [^18^F]F13640 Binding in Cerebellum

A pronounced sensitivity of [^18^F]F13640 binding in the cerebellum to fenfluramine administration was observed in the PET experiments. This can be puzzling considering the high uptake of [^18^F]F13640 in the cortical parts and the highest amplitude of effects in pretreatment and challenge paradigm. These *in vivo* data are also in discordance with our *in vitro* autoradiography results in the same region, i.e., a low level of binding which is not affected by serotonin displacement and not influenced by addition of Gpp(NH)p in buffer. However, for other regions, we observed the same range of decrease produced by uncoupling of receptors as described for the D2/D3 agonist [^18^F]-5-OH-FPPAT, an average of 50% (Mukherjee et al., 2017). Classical 5-HT_1A_ radiotracers also show low specific binding in the cerebellum (Mathis et al., 1994; Shiue et al., 1997). However, the present *in vivo* results must be compared with previous data revealing decreased [^18^F]F13640 binding in the cerebellum after pre-administration of a 5-HT_1A_ antagonist or agonist, indicating the existence of 5-HT_1A_ receptors in this brain region (Vidal et al., 2018a). Furthermore, 5-HT release could occur in this region given that cat cerebellum contains 5-HT neuronal projections (Kitzman and Bishop, 1994; Leger et al., 2001). In this regard, 5-HT iontophoresis in cerebellar gray matter in cat induces strong inhibitory effects on local neurons – these are mediated by 5-HT_1A_ receptors, highlighting the functional importance of cerebellar 5-HT_1A_ receptors of this species (Kerr and Bishop, 1991). An autoradiographic study using a radiolabeled SSRI, [^3^H]paroxetine, also indicates that 5-HT transporter expression occurs in the cat cerebellum, both in the granular layer and the dentate nucleus (Charnay et al., 1997). In this context of cerebellar heterogeneity of 5-HT_1A_ receptor density, and strong effects of d-fenfluramine in the gray matter, we thus did not consider the cerebellar nuclei as a reference region. Instead, we identified a region in the anterior part of the white matter centrum semiovale that displayed consistently low signal in the three conditions and was less likely to be impacted by partial volume effects (Figure 4). This region was therefore used as reference, similarly to a previous study (Kudomi et al., 2013), for estimation of binding ratios and for the lp-ntPET modeling approach.

### Kinetic Modeling of Serotonin Release

The kinetic modeling approach used in this study calculated that fenfluramine-induced serotonin release caused an increase of 10–30% of receptor occupancy by serotonin (Figure 6). We observed a delay of 45 min between d-fenfluramine administration and the maximal occupancy effect. Furthermore, we noticed that modeled curves of serotonin release were relatively close in the four cats. The variability observed is mainly driven by the maximal amplitude of each curve and corresponds to 8–15% across individuals, which can be considered as a low inter-subject variability and is in the same range as the variability obtained with binding ratios in the challenge study. Considering the variability of lp-ntPET model described in Irace et al. (2020), the results of discharge modeling in the article are very close between the four cats and could probably be reduced even more by using the robust b-ntPET algorithm presented by Irace et al. (2020). Finally, the kinetic modeling results suggest an apparent slow 5-HT release after d-fenfluramine injection, which is unexpected given its fast kinetics observed using microdialysis experiments. In fact, the slow washout of [^18^F]F13640 seems to be responsible for this difference. Furthermore, results must be analyzed with caution because anesthesia is considered to change endogenous levels of 5-HT (Yokoyama et al., 2016) and numerous studies on conscious animals reveals differences in 5-HT levels or radiotracer binding (Zimmer et al., 2002; Udo de Haes et al., 2005; Yokoyama et al., 2016).

In the absence of microdialysis, the lp-ntPET model provides the first modeling of serotonin release induced by d-fenfluramine with a PET radiotracer of serotonin receptors. We proposed a lp-ntPET model which successfully discriminates control and challenge condition and confirms the hypothesis of a tracer displacement by a significant release of serotonin. The short acquisition time of 90 min appears as the major limitation of our study given the slow wash-out of the radiotracer (Vidal et al., 2018a). However, these results confirm that [^18^F]F13640 binding is reversible, despite slow kinetics, by demonstrating its sensitivity to serotonin release. Further studies using [^18^F]F13640 will need to be carried out using longer time scans to improve estimation of serotonin occupancy.

## Conclusion

In conclusion, as compared with previous antagonist or partial agonist radiotracers, the present results demonstrate a high sensitivity of [^18^F]F13640 to serotonin release induced by d-fenfluramine. Although we used a low dose of d-fenfluramine (1 mg/kg), a significant displacement of [^18^F]F13640 was observed for both pretreatment and challenge studies. These results support our initial hypothesis that an agonist PET tracer is better adapted to measure endogenous levels of serotonin than antagonist PET tracers. Indeed, while the agonist PET tracer, [^18^F]F13640, binds only to G-protein-coupled 5-HT_1A_ receptors, in the same manner as the endogenous neurotransmitter, antagonists binds to both G-protein-coupled and uncoupled receptors. Consequently, antagonist PET tracers appear less effective to detect changes in serotonin levels, especially when G-protein-coupled receptors constitute only a small proportion of the total receptor population (Shiue et al., 1997). Finally, the first kinetic modeling of serotonin release using [^18^F]F13640 as radiotracer demonstrates the feasibility of evaluating 5-HT release in displacement experiments. The slow kinetics of our radiotracer indicate the necessity to perform longer acquisition times (or delayed PET scans) to ensure a better understanding of displacement or pretreatment studies, but also enable to perform displacement experiments without using a bolus followed by constant infusion administration. Overall, these results support the use of [^18^F]F13640 to investigate changes in serotonin levels in humans, particularly in the context of experimental paradigms involving physiological (sleep/wake states) or pathological (neuropsychiatric pathologies) fluctuations of extracellular serotonin.

## Data Availability Statement

The original contributions presented in the study are included in the article, further inquiries can be directed to the corresponding author.

## Ethics Statement

The animal study was reviewed and approved by the Celyne (CEEA-42), Lyon, France.

## Author Contributions

MC and BV carried out the experiments with PET imaging, performed the data analysis, participated in the study design, and co-wrote the manuscript. SF, FL, and CB carried out the experiments with PET imaging. TI and TB carried out the radiosynthesis of [^18^F]F13640. JR, NC, IM, and ZI analyzed the PET data. AN-T provided precursor of [^18^F]F13640 and reviewed the manuscript. LZ initiated the study, participated in its design, and reviewed the manuscript. All authors read and approved the final manuscript.

## Conflict of Interest

AN-T is employed by Neurolixis. The remaining authors declare that the research was conducted in the absence of any commercial or financial relationships that could be construed as a potential conflict of interest.

## References

[B1] Akimova E. Lanzenberger R. Kasper S. (2009). The serotonin-1A receptor in anxiety disorders. *Biol. Psychiatry* 66 627–635. 10.1016/j.biopsych.2009.03.012 19423077

[B2] Assié M. B. Koek W. (1996). Possible in vivo 5-HT reuptake blocking properties of 8-OH-DPAT assessed by measuring hippocampal extracellular 5-HT using microdialysis in rats. *Br. J. Pharmacol.* 119 845–850. 10.1111/j.1476-5381.1996.tb15749.x 8922730 PMC1915946

[B3] Aznavour N. Rbah L. Riad M. Reilhac A. Costes N. Descarries L. (2006). A PET imaging study of 5-HT1A receptors in cat brain after acute and chronic fluoxetine treatment. *NeuroImage* 33 834–842. 10.1016/j.neuroimage.2006.08.012 16996750

[B4] Balcioglu A. Wurtman R. J. (1998). Effects of fenfluramine and phentermine (fen-phen) on dopamine and serotonin release in rat striatum: in vivo microdialysis study in conscious animals. *Brain Res.* 813 67–72. 10.1016/s0006-8993(98)01003-8 9824670

[B5] Bantick R. A. Rabiner E. A. Hirani E. de Vries M. H. Hume S. P. Grasby P. M. (2004). Occupancy of agonist drugs at the 5-HT1A receptor. *Neuropsychopharmacology* 29 847–859. 10.1038/sj.npp.1300390 14985704

[B6] Buhot M. C. (1997). Serotonin receptors in cognitive behaviors. *Curr. Opin. Neurobiol.* 7 243–254. 9142756 10.1016/s0959-4388(97)80013-x

[B7] Charnay Y. Léger L. Vallet P. Greggio B. Hof P. R. Jouvet M. (1997). Autoradiographic distribution of [3H]paroxetine binding sites in the cat brain. *Biogenic Amines* 13 39–54.

[B8] Colom M. Costes N. Redouté J. Dailler F. Gobert F. Le Bars D. (2020). 18F-F13640 PET imaging of functional receptors in humans. *Eur. J. Nucl. Med. Mol. Imaging* 47 220–221. 10.1007/s00259-019-04473-7 31414208

[B9] Colom M. Vidal B. Zimmer L. (2019). Is there a role for GPCR agonist radiotracers in PET neuroimaging? *Front. Mol. Neurosci.* 12:255. 10.3389/fnmol.2019.00255 31680859 PMC6813225

[B10] da Cunha-Bang S. Ettrup A. Mc Mahon B. Skibsted A. P. Schain M. Lehel S. (2019). Measuring endogenous changes in serotonergic neurotransmission with [11C]Cimbi-36 positron emission tomography in humans. *Transl. Psychiatry* 9:134. 10.1038/s41398-019-0468-8 30975977 PMC6459901

[B11] Erritzoe D. Ashok A. H. Searle G. E. Colasanti A. Turton S. Lewis Y. (2020). Serotonin release measured in the human brain: a PET study with [11C]CIMBI-36 and d-amphetamine challenge. *Neuropsychopharmacology* 45 804–810. 10.1038/s41386-019-0567-5 31715617 PMC7075951

[B12] Finnema S. J. Varrone A. Hwang T.-J. Halldin C. Farde L. (2012). Confirmation of fenfluramine effect on 5-HT(1B) receptor binding of [(11)C]AZ10419369 using an equilibrium approach. *J. Cereb. Blood Flow Metab. Off. J. Int. Soc. Cereb. Blood Flow Metab.* 32 685–695. 10.1038/jcbfm.2011.172 22167236 PMC3318146

[B13] Gardier A. M. Trillat A.-C. Malagié I. Jacquot C. (1994). 8-OH-DPAT attenuates the dexfenfluramine-induced increase in extracellular serotonin: an in vivo dialysis study. *Eur. J. Pharmacol.* 265 107–110. 10.1016/0014-2999(94)90231-3 7883022

[B14] Ginovart N. (2005). Imaging the dopamine system with in vivo [11C]raclopride displacement studies: understanding the true mechanism. *Mol. Imaging Biol. MIB Off. Publ. Acad. Mol. Imaging* 7 45–52. 10.1007/s11307-005-0932-0 15912275

[B15] Heusler P. Palmier C. Tardif S. Bernois S. Colpaert F. C. Cussac D. (2010). [3H]-F13640, a novel, selective and high-efficacy serotonin 5-HT1A receptor agonist radioligand. *Naunyn Schmiedebergs Arch. Pharmacol.* 382 321–330. 10.1007/s00210-010-0551-4 20799027

[B16] Hume S. Hirani E. Opacka-Juffry J. Myers R. Townsend C. Pike V. (2001). Effect of 5-HT on binding of [(11)C] WAY 100635 to 5-HT(IA) receptors in rat brain, assessed using in vivo microdialysis nd PET after fenfluramine. *Synapse* 41 150–159. 10.1002/syn.1069 11400181

[B17] Irace Z. Mérida I. Redouté J. Fonteneau C. Suaud-Chagny M.-F. Brunelin J. (2020). Bayesian estimation of the ntPET model in single-scan competition PET studies. *Front. Physiol.* 11:498. 10.3389/fphys.2020.00498 32508679 PMC7248280

[B18] Jagoda E. M. Lang L. Tokugawa J. Simmons A. Ma Y. Contoreggi C. (2006). Development of 5-HT1A receptor radioligands to determine receptor density and changes in endogenous 5-HT. *Synapse* 59 330–341. 10.1002/syn.20246 16440292

[B19] Jørgensen L. M. Weikop P. Villadsen J. Visnapuu T. Ettrup A. Hansen H. D. (2017). Cerebral 5-HT release correlates with [11C]Cimbi36 PET measures of 5-HT2A receptor occupancy in the pig brain. *J. Cereb. Blood Flow Metab. Off. J. Int. Soc. Cereb. Blood Flow Metab.* 37 425–434. 10.1177/0271678X16629483 26825776 PMC5381441

[B20] Kerr C. W. H. Bishop G. A. (1991). Topographical organization in the origin of serotoninergic projections to different regions of the cat cerebellar cortex. *J. Comp. Neurol.* 304 502–515. 10.1002/cne.903040313 2022761

[B21] Kitzman P. H. Bishop G. A. (1994). The origin of serotoninergic afferents to the cat’s cerebellar nuclei. *J. Comp. Neurol.* 340 541–550. 10.1002/cne.903400407 8006216

[B22] Kristian Eide P. Mjellem Joly N. Hole K. (1990). The role of spinal cord 5-HT1A and 5-HT1B receptors in the modulation of a spinal nociceptive reflex. *Brain Res.* 536 195–200. 10.1016/0006-8993(90)90025-7 2150769

[B23] Kudomi N. Hirano Y. Koshino K. Hayashi T. Watabe H. Fukushima K. (2013). Rapid quantitative *CBF* and *CMRO* _2_ measurements from a single *PET* scan with sequential administration of dual ^15^ O-Labeled tracers. *J. Cereb. Blood Flow Metab.* 33 440–448. 10.1038/jcbfm.2012.188 23232945 PMC3587817

[B24] Kyme A. Z. Angelis G. I. Eisenhuth J. Fulton R. R. Zhou V. Hart G. (2019). Open-field PET: simultaneous brain functional imaging and behavioural response measurements in freely moving small animals. *NeuroImage* 188 92–101. 10.1016/j.neuroimage.2018.11.051 30502443

[B25] Lancelot S. Costes N. Lemoine L. Zimmer L. (2010). Development and evaluation of a digital atlas for PET neuroimaging in domestic cat (*Felis catus*). *Eur. J. Nucl. Med. Mol. Imaging* 37 S387–S387.

[B26] Laruelle M. (2000). Imaging synaptic neurotransmission with in vivo binding competition techniques: a critical review. *J. Cereb. Blood Flow Metab.* 20 423–451. 10724107 10.1097/00004647-200003000-00001

[B27] Leger L. Charnay Y. Hof P. R. Bouras C. Cespuglio R. (2001). Anatomical distribution of serotonin-containing neurons and axons in the central nervous system of the cat. *J. Comp. Neurol.* 433 157–182. 11283957

[B28] Lemoine L. Becker G. Vacher B. Billard T. Lancelot S. Newman-Tancredi A. (2012). Radiosynthesis and preclinical evaluation of 18F-F13714 as a fluorinated 5-HT1A receptor agonist radioligand for PET neuroimaging. *J. Nucl. Med.* 53 969–976. 10.2967/jnumed.111.101212 22577236

[B29] Lemoine L. Verdurand M. Vacher B. Blanc E. Le Bars D. Newman-Tancredi A. (2010). [18F]F15599, a novel 5-HT1A receptor agonist, as a radioligand for PET neuroimaging. *Eur. J. Nucl. Med. Mol. Imaging* 37 594–605. 10.1007/s00259-009-1274-y 19789870

[B30] Maćkowiak M. Czyrak A. Wedzony K. (2000). [The involvement of 5-HT1a serotonin receptors in the pathophysiology and pharmacotherapy of schizophrenia]. *Psychiatr. Pol.* 34 607–621. 11059260

[B31] Mathis C. A. Simpson N. R. Mahmood K. Kinahan P. E. Mintun M. A. (1994). [11C]WAY 100635: a radioligand for imaging 5-HT1A receptors with positron emission tomography. *Life Sci.* 55 L403–L407. 10.1016/0024-3205(94)00324-6 7968222

[B32] Merida I. Olivier F. Hammers A. Redouté J. Reilhac A. Costes N. (2018). “Kinetic modelling for endogenous neurotransmitter discharge characterization using PET imaging: optimization of lp-ntPET,” in *Proceedings of the 8th International Symposium of Functional Neuroreceptor Mapping of the Living Brain (NRM18)*, London.

[B33] Milak M. S. DeLorenzo C. Zanderigo F. Prabhakaran J. Kumar J. S. D. Majo V. J. (2010). In vivo quantification of human serotonin 1A receptor using 11C-CUMI-101, an agonist PET radiotracer. *J. Nucl. Med.* 51 1892–1900. 10.2967/jnumed.110.076257 21098796 PMC3856257

[B34] Miquel M. C. Doucet E. Riad M. Adrien J. Vergé D. Hamon M. (1992). Effect of the selective lesion of serotoninergic neurons on the regional distribution of 5-HT1A receptor mRNA in the rat brain. *Brain Res. Mol. Brain Res.* 14 357–362. 1326699 10.1016/0169-328x(92)90104-j

[B35] Mukherjee J. Majji D. Kaur J. Constantinescu C. C. Narayanan T. K. Shi B. (2017). PET radiotracer development for imaging high-affinity state of dopamine D2 and D3 receptors: binding studies of fluorine-18 labeled aminotetralins in rodents: MUKHERJEE ET AL. *Synapse* 71:e21950. 10.1002/syn.21950 27864853 PMC5363407

[B36] Nénonéné E. K. Radja F. Carli M. Grondin L. Reader T. A. (1994). Heterogeneity of cortical and hippocampal 5-HT1A receptors: a reappraisal of homogenate binding with 8-[3H]hydroxydipropylaminotetralin. *J. Neurochem.* 62 1822–1834. 10.1046/j.1471-4159.1994.62051822.x 8158133

[B37] Nichols D. E. Nichols C. D. (2008). Serotonin receptors. *Chem. Rev.* 108 1614–1641. 18476671 10.1021/cr078224o

[B38] Normandin M. D. Schiffer W. K. Morris E. D. (2012). A linear model for estimation of neurotransmitter response profiles from dynamic PET data. *NeuroImage* 59 2689–2699. 10.1016/j.neuroimage.2011.07.002 21767654 PMC3702051

[B39] Paterson L. M. Tyacke R. J. Nutt D. J. Knudsen G. M. (2010). Measuring endogenous 5-HT release by emission tomography: promises and pitfalls. *J. Cereb. Blood Flow Metab.* 30 1682–1706. 10.1038/jcbfm.2010.104 20664611 PMC3023404

[B40] Pinborg L. H. Feng L. Haahr M. E. Gillings N. Dyssegaard A. Madsen J. (2012). No change in [11C]CUMI-101 binding to 5-HT1A receptors after intravenous citalopram in human. *Synapse* 66 880–884. 10.1002/syn.21579 22730164

[B41] Portas C. M. McCarley R. W. (1994). Behavioral state-related changes of extracellular serotonin concentration in the dorsal raphe nucleus: a microdialysis study in the freely moving cat. *Brain Res.* 648 306–312. 10.1016/0006-8993(94)91132-0 7922546

[B42] Radja F. Laporte A. M. Daval G. Vergé D. Gozlan H. Hamon M. (1991). Autoradiography of serotonin receptor subtypes in the central nervous system. *Neurochem. Int.* 18 1–15. 20504669 10.1016/0197-0186(91)90029-d

[B43] Riad M. Garcia S. Watkins K. C. Jodoin N. Doucet E. Langlois X. (2000). Somatodendritic localization of 5-HT1A and preterminal axonal localization of 5-HT1B serotonin receptors in adult rat brain. *J. Comp. Neurol.* 417 181–194. 10660896

[B44] Richardson-Jones J. W. Craige C. P. Guiard B. P. Stephen A. Metzger K. L. Kung H. F. (2010). 5-HT1A autoreceptor levels determine vulnerability to stress and response to antidepressants. *Neuron* 65 40–52. 10.1016/j.neuron.2009.12.003 20152112 PMC2941196

[B45] Sander C. Y. Hooker J. M. Catana C. Normandin M. D. Alpert N. M. Knudsen G. M. (2013). Neurovascular coupling to D2/D3 dopamine receptor occupancy using simultaneous PET/functional MRI. *Proc. Natl. Acad. Sci. U.S.A.* 110 11169–11174. 10.1073/pnas.1220512110 23723346 PMC3703969

[B46] Selvaraj S. Turkheimer F. Rosso L. Faulkner P. Mouchlianitis E. Roiser J. P. (2012). Measuring endogenous changes in serotonergic neurotransmission in humans: a [11C]CUMI-101 PET challenge study. *Mol. Psychiatry* 17 1254–1260. 10.1038/mp.2012.78 22665264

[B47] Shimizu S. Ohno Y. (2013). Improving the treatment of Parkinson’s disease: a novel approach by modulating 5-HT(1A) receptors. *Aging Dis.* 4 1–13. 23423244 PMC3570136

[B48] Shiue C. Y. Shiue G. G. Mozley P. D. Kung M. P. Zhuang Z. P. Kim H. J. (1997). P-[18F]-MPPF: a potential radioligand for PET studies of 5-HT1A receptors in humans. *Synapse* 25 147–154. 9021895 10.1002/(SICI)1098-2396(199702)25:2<147::AID-SYN5>3.0.CO;2-C

[B49] Truchot L. Costes S. N. Zimmer L. Laurent B. Le Bars D. Thomas-Anterion C. (2007). Up-regulation of hippocampal serotonin metabolism in mild cognitive impairment. *Neurology* 69 1012–1017. 10.1212/01.wnl.0000271377.52421.4a 17785670

[B50] Tyacke R. J. Nutt D. J. (2015). Optimising PET approaches to measuring 5-HT release in human brain. *Synapse* 69 505–511. 10.1002/syn.21835 26089243

[B51] Udo de Haes J. I. Cremers T. I. F. H. Bosker F.-J. Postema F. Tiemersma-Wegman T. D. den Boer J. A. (2005). Effect of increased serotonin levels on [18F]MPPF binding in rat brain: fenfluramine vs the combination of citalopram and ketanserin. *Neuropsychopharmacol. Off. Publ. Am. Coll. Neuropsychopharmacol.* 30 1624–1631. 10.1038/sj.npp.1300721 15827572

[B52] Udo de Haes J. I. Harada N. Elsinga P. H. Maguire R. P. Tsukada H. (2006). Effect of fenfluramine-induced increases in serotonin release on [18F]MPPF binding: a continuous infusion PET study in conscious monkeys. *Synapse* 59 18–26. 10.1002/syn.20209 16237679

[B53] Verge D. Daval G. Patey A. Gozlan H. el Mestikawy S. Hamon M. (1985). Presynaptic 5-HT autoreceptors on serotonergic cell bodies and/or dendrites but not terminals are of the 5-HT1A subtype. *Eur. J. Pharmacol.* 113 463–464. 10.1016/0014-2999(85)90099-8 2931289

[B54] Vidal B. Fieux S. Colom M. Billard T. Bouillot C. Barret O. (2018a). 18F-F13640 preclinical evaluation in rodent, cat and primate as a 5-HT1A receptor agonist for PET neuroimaging. *Brain Struct. Funct.* 223 2973–2988. 10.1007/s00429-018-1672-7 29730825

[B55] Vidal B. Fieux S. Redouté J. Villien M. Bonnefoi F. Le Bars D. (2018b). In vivo biased agonism at 5-HT1A receptors: characterisation by simultaneous PET/MR imaging. *Neuropsychopharmacology* 43 2310–2319. 10.1038/s41386-018-0145-2 30030540 PMC6135772

[B56] Yang K.-C. Takano A. Halldin C. Farde L. Finnema S. J. (2018). Serotonin concentration enhancers at clinically relevant doses reduce [11C]AZ10419369 binding to the 5-HT1B receptors in the nonhuman primate brain. *Transl. Psychiatry* 8:132. 10.1038/s41398-018-0178-7 30013068 PMC6048172

[B57] Yokoyama C. Mawatari A. Kawasaki A. Takeda C. Onoe K. Doi H. (2016). Marmoset serotonin 5-HT _1A_ receptor mapping with a biased agonist PET Probe ^18^ F-F13714: comparison with an antagonist tracer ^18^ F-MPPF in awake and anesthetized states. *Int. J. Neuropsychopharmacol.* 19:yw079. 10.1093/ijnp/pyw079 27608810 PMC5203761

[B58] Zhang Y. Fox G. B. (2012). PET imaging for receptor occupancy: meditations on calculation and simplification. *J. Biomed. Res.* 26 69–76. 10.1016/S1674-8301(12)60014-1 23554733 PMC3597321

[B59] Zimmer L. Mauger G. Le Bars D. Bonmarchand G. Luxen A. Pujol J.-F. (2002). Effect of endogenous serotonin on the binding of the 5-hT1A PET ligand 18F-MPPF in the rat hippocampus: kinetic beta measurements combined with microdialysis. *J. Neurochem.* 80 278–286. 10.1046/j.0022-3042.2001.00696.x 11902118

[B60] Zimmer L. Rbah L. Giacomelli F. Le Bars D. Renaud B. (2003). A reduced extracellular serotonin level increases the 5-HT1A PET ligand 18F-MPPF binding in the rat hippocampus. *J. Nucl. Med. Off. Publ. Soc. Nucl. Med.* 44 1495–1501. 12960198

[B61] Zimmer L. Riad M. Rbah L. Belkacem-Kahlouli A. Le Bars D. Renaud B. (2004). Toward brain imaging of serotonin 5-HT1A autoreceptor internalization. *NeuroImage* 22 1421–1426. 10.1016/j.neuroimage.2004.03.020 15219613

